# The Interplay of Dyslipidemia, Oxidative Stress, and Clinical Outcomes in Acute Ischemic Stroke Patients with and without Coronary Artery Disease

**DOI:** 10.3390/biomedicines12020332

**Published:** 2024-02-01

**Authors:** Branislav Kollar, Pavel Siarnik, Katarina Konarikova, Stanislav Oravec, Stanislava Klobucka, Katarina Klobucnikova, Michal Poddany, Zofia Radikova, Maria Janubova, Peter Turcani, Livia Gajdosova, Ingrid Zitnanova

**Affiliations:** 1 1st Department of Neurology, Faculty of Medicine, Comenius University, 813 69 Bratislava, Slovakia; kollar3@uniba.sk (B.K.); klobucnikova@gmail.com (K.K.); peter.turcani@sm.unb.sk (P.T.); 2 Institute of Medical Chemistry, Biochemistry and Clinical Biochemistry, Faculty of Medicine, Comenius University, 813 72 Bratislava, Slovakia; katarina.konarikova@fmed.uniba.sk (K.K.); maria.janubova@fmed.uniba.sk (M.J.); livia.gajdosova@fmed.uniba.sk (L.G.); ingrid.zitnanova@fmed.uniba.sk (I.Z.); 3 1st Department of Internal Medicine, Faculty of Medicine, Comenius University, 813 69 Bratislava, Slovakia; stanislavoravec@yahoo.com; 4 Rehabilitation Centre Harmony, 841 01 Bratislava, Slovakia; stanislavaklobucka@gmail.com; 5 Department of Neurology, General Hospital, 031 01 Liptovsky Mikulas, Slovakia; michal.poddany@gmail.com; 6 Institute of Clinical and Translational Research, Biomedical Research Center, Slovak Academy of Sciences, 845 05 Bratislava, Slovakia; zofia.radikova@savba.sk

**Keywords:** ischemic stroke, coronary artery disease, lipoproteins, oxidative stress, lipoperoxides

## Abstract

We assessed lipid and lipoprotein profiles, along with oxidative stress (OS) parameters, in patients within the crucial 24 h period following an acute ischemic stroke (AIS), comparing those with and without coronary artery disease (CAD). We aimed to correlate these measures with clinical condition scales (NIHSS, mRS) post-AIS. This study included 27 AIS patients without CAD (AIS group) and 37 AIS patients with CAD (CAD-AIS group). Using polyacrylamide gel electrophoresis (Lipoprint system), we determined plasma LDL and HDL subfractions. Spectrophotometric methods were used to assess plasma antioxidant capacity, lipoperoxides, homocysteine (HC) levels, paraoxonase1, and catalase activities. We also measured urine isoprostanes and the activities of antioxidant enzymes (SOD, GPx) with commercial kits. CAD-AIS patients had notably higher HC levels, while there were no significant differences in lipoprotein subfractions and OS parameters between both groups. In the AIS group, mRS scores showed negative correlations with catalase, GPx activities, and total cholesterol. In the CAD-AIS group, atherogenic lipoproteins (IDLC, LDL2, LDL3–7) exhibited a significant positive correlation with mRS. This study underscores the role of dyslipidemia and OS in the development of AIS and CAD. It emphasizes the complex connections between specific biomarkers and post-stroke clinical outcomes. Our results suggest a significant impact of CAD treatment on lipid profile but not on homocysteine levels. The traditional narrative associating high cholesterol as the ultimate risk factor for cardiovascular diseases needs to be challenged, at least with respect to neurological outcomes. These insights may guide more targeted therapeutic approaches.

## 1. Introduction

Acute ischemic stroke (AIS) is the second leading cause of death worldwide and a significant contributor to disability [1]. It is one of the most common subtypes of stroke, and approximately one in four AIS patients also have coronary artery disease (CAD) [2]. Coronary artery disease is characterized by reduced blood flow to the heart muscle caused by the narrowing or blockage of coronary arteries due to cholesterol deposits. Atherosclerosis, a progressive inflammatory disease of the arteries, is the most common underlying cause of CAD. Various epidemiological studies have linked elevated low-density lipoprotein cholesterol (LDL-C) levels to an increased risk of developing CAD [3,4]. Consequently, LDL-C remains the primary treatment target in major guidelines for both primary and secondary prevention [5].

However, it has been observed that many individuals who develop CAD have LDL-C levels within the normal range [6]. This finding challenges the traditional approach of using LDL-C concentrations as the primary lipid target in managing CAD risk and has led to the search for new risk factors of coronary events [7]. One potential factor is lipoprotein subfractions with different density, size, and atherogenic profiles. The Lipoprint system, utilizing polyacrylamide gel electrophoresis (Quantimetrix Corp., Redondo Beach, CA, USA), can divide blood lipoproteins into smaller subfractions, some of which may not pose an atherogenic risk. This study’s Lipoprint system identifies 7 LDL- and 10 HDL-cholesterol subfractions. LDL subfractions are categorized into large (subfractions 1–2) and small, dense LDL subfractions (subfractions 3–7), while HDL subfractions are divided into large (subfractions 1–3), medium (subfractions 4–7), and small (subfractions 8–10) [8,9]. It is supposed that small LDL3–7 and small HDL8–10 subfractions represent the atherogenic part of the lipoprotein spectrum due to their low recognition by cell receptors and their ability to easily penetrate into the subendothelial space, where they can form cholesterol deposits [9].

Oxidative stress plays a crucial role in the pathogenesis of cardiovascular diseases [10]. Several studies have reported higher levels of oxidative stress markers, such as malondialdehyde (MDA), reactive oxygen species (ROS), and oxidized low-density lipoproteins (oxLDL), in patients following an acute ischemic stroke [11,12].

Paraoxonase 1 (PON1) activity, examined in this study, is one of the enzymes linked to oxidative stress, oxidized lipids, and the metabolism of oxidized low-density lipoproteins (oxLDL). PON1 is a polymorphic enzyme associated with high-density lipoproteins (HDLs) that provides antioxidant and anti-inflammatory functions [13]. Two common polymorphisms in the PON1 gene, Q192R and L55M substitutions, determine the inter-individual variation in PON1 activity. The association of these polymorphisms with the risk of ischemic stroke remains controversial [14].

PON1 activity is reduced in stroke patients, which significantly correlates inversely with carotid and cerebral atherosclerosis. The presence of the R allele of the Q192R PON1 polymorphism seems to potentiate this risk for stroke [15]. PON1 is a potential player in the pathogenesis of several neurological disorders, and more research is warranted to ascertain the precise pathogenic links and the prognostic value of its activity [15].

To date, there has been no published study investigating the effect of CAD on lipoprotein subfractions and oxidative stress markers in patients within the critical 24 h window following an acute ischemic stroke. Our study aimed to explore the potential influence of lipid parameters on the clinical outcomes in ischemic stroke patients suffering from CAD. The findings from this study could have implications for better risk stratification and management strategies for individuals with coexisting CAD and AIS.

## 2. Materials and Methods

### 2.1. Study Population

Sixty-four consecutive patients within 24 h after an acute ischemic stroke (AIS) were included in our study. To determine the subtype of stroke, a clinical examination followed by a CT scan of the brain was performed. Data on admissions for AIS were collected at the 1st Department of Neurology, Faculty of Medicine, Comenius University, Bratislava, Slovakia. These data included routine hematological and biochemical parameters, as well as vascular risk factors such as age, gender, and current treatment with antihypertensive therapy and antidiabetic, antithrombic, anticoagulation, or lipid-lowering agents. Coronary artery disease was defined as the presence of myocardial infarction, angina pectoris, or severe stenotic changes in the coronary arteries identified by angiography or computed tomography [16]. A total of 27 patients (20 males and 7 females) (mean age 63.89 ± 13.83 years) were patients without CAD (group AIS), and there were 37 patients with CAD (15 males and 22 females) (mean age 77.8 1 ± 14.41 years) (group CAD-AIS). Blood and urine samples were collected from all study participants. This study was approved by the local ethics committee. All participants in our study signed informed consent.

### 2.2. Plasma Samples

To obtain blood plasma, fasting venous blood with EDTA anticoagulant was collected from each patient within 24 h after AIS. Blood samples were centrifuged for 5 min at 1200× *g* and at 4 °C, aliquoted, stored at −80 °C, and used for determination of the lipid profile and oxidative stress parameters.

### 2.3. Hemolysate Samples

To prepare hemolysates, erythrocytes (0.5 mL) were washed three times with physiological solution (5 mL 0.9% NaCl) and centrifuged at 660× *g* for 5 min at 4 °C with subsequent hemolysis in chilled distilled water. Hemolysates were stored at −80 °C and used for the determination of activities of antioxidant enzymes.

### 2.4. Urine Samples

Overnight fasting urine samples were stored in aliquots at −80 °C.

### 2.5. Lipid Profile

Total cholesterol (TC), HDL cholesterol, LDL cholesterol, triacylglycerols (TAGs) in plasma, and creatinine in urine were determined in a certified laboratory by the enzymatic method (Roche Diagnostics, Mannheim, Germany).

### 2.6. Quantitative Analysis of Lipoprotein Subfractions

VLDL cholesterol and subfractions of low-density lipoproteins (LDL) and high-density lipoproteins (HDL) were determined in plasma using the Lipoprint LDL system (Quantimetrix Corp., Redondo Beach, CA, USA) according to the manufacturer’s instructions.

### 2.7. NIHSS

The level of clinical disability of patients after AIS was evaluated using the National Institutes of Health Stroke Scale (NIHSS), which measures neurological functions in patients with signs and symptoms of AIS. The worse the patient’s disability, the higher the NIHSS value [17]. The minimum score on the NIHSS is 0, which indicates no neurological deficits. The maximum score on the NIHSS is 42, which indicates severe neurological deficits across all evaluated functions.

### 2.8. The Modified Rankin Scale (mRS)

The modified Rankin Scale (mRS) is a commonly used clinical tool to assess the functional outcomes and disability levels of stroke patients. It is the scale ranging from 0 to 6 that provides a standardized way to evaluate the overall disability and dependency of a patient after a stroke or other neurological problems [17]. The higher mRS scores indicate more severe disability in stroke patients.

### 2.9. Oxidative Stress Markers

Lipid peroxides in plasma samples were assessed using a UV-1800 Shimadzu Spectrophotometer. This was carried out spectrophotometrically based on the method described by El-Saadani et al. (1989) [18]. Another marker of lipid oxidation, isoprostanes (8-iso prostaglandin F2α), in urine, was determined using a commercially available EIA kit from Cayman Chemical, USA, following the provided instructions.

To detect protein carbonyls in plasma, a commercial OxiSelectTM protein carbonyl ELISA kit from Cell Biolabs, USA, was utilized according to the manufacturer’s instructions. The TEAC assay by Re et al. was employed to measure the plasma antioxidant capacity [19]. Trolox, a synthetic water-soluble form of vitamin E, was used as the reference antioxidant.

For assessing paraoxonase activity (PON1) in plasma, spectrophotometry was used with phenylacetate as a substrate, following the method by Gan et al. [20]. The enzyme activity was expressed in U/mL, using a molar extinction coefficient of 1310 mol^−1^·L·cm^−1^, where 1 U corresponds to 1 μmol of phenol produced per minute. 

To determine the superoxide dismutase (SOD) activity in erythrocyte lysates, the SOD Assay kit from Sigma-Aldrich Co., St. Louis, MO, USA, was employed. The SOD activity was expressed in U/mg hemoglobin, where 1 U of SOD activity inhibits the rate of chromagen reduction by 50%.

Catalase activity in erythrocytes was determined following the method by Bergmeyer [21]. Additionally, GPx activity in hemolysates was assessed using a commercial kit from Cayman Chemical, Ann Arbor, MI, USA, following the manufacturer’s protocol. Each sample was analyzed in triplicate.

Hemoglobin concentration in the erythrocyte lysates was measured using Drabkin’s reagent [22].

### 2.10. Statistical Analysis

Statistical analyses were performed using SPSS version 18 (SPSS Inc., Chicago, IL, USA). Normally distributed data are presented as means ± standard deviation (SD), while not normally distributed data are presented as medians with interquartile range (IQR), representing minimal and maximal values. To compare groups, the Chi-squared test, Student *t*-test, or the Mann–Whitney U test was applied to specific variables. A *p*-value < 0.05 was considered statistically significant. Pearson’s or Spearman’s correlation coefficients were used to determine the relationships between individual parameters.

## 3. Results

The plasma lipid profile of patients within 24 h after AIS with/without CAD is shown in Table 1. The patient group within 24 h after AIS with coronary artery disease (CAD-AIS) exhibited significantly lower total cholesterol (TC), very low-density lipoprotein cholesterol (VLDL), low-density cholesterol (LDL), and triacylglycerol (TAG) levels compared to AIS group without CAD. However, levels of homocysteine (HC) were significantly higher in CAD-AIS patients compared to AIS patients without CAD.

Patients in the AIS group with coexisting CAD were found to be significantly older (*p* < 0.001) in comparison to those without CAD when experiencing the AIS event (Table 1). Furthermore, a significantly higher proportion of individuals in the CAD-AIS group had previously suffered from another episode of AIS, as opposed to participants in the AIS group without CAD.

Table 1 illustrates no significant differences between both study groups in parameters such as the National Institutes of Health Stroke Scale (NIHSS) score or modified Rankin Scale (mRS) score. However, significant differences were found in the usage of some therapeutic interventions between the CAD-AIS and AIS groups.

To find out which subfractions are changed in different lipoprotein fractions, we used a polyacrylamide gel electrophoresis (Lipoprint LDL and HDL System; Quantimetrix, Redondo Beach, CA, USA). However, we found no statistically significant differences in lipoprotein subfractions between both groups of patients (Table 2).

Our results presented in Table 3 compare the levels of oxidative stress markers in patients with coronary artery disease after an acute ischemic stroke (CAD-AIS) and patients without coronary artery disease after an acute ischemic stroke (AIS).

Our results show some variations in oxidative stress markers between the two groups (CAD-AIS and AIS), but none of these differences are statistically significant. However, some parameters (lipoperoxides, protein carbonyls, and activity of paraoxonase A) have *p*-values that are close to being statistically significant, indicating a possible trend that warrants further investigation with a larger sample size.

We also investigated correlations between the clinical condition of patients within 24 h after an ischemic stroke, as measured by the National Institutes of Health Stroke Scale (NIHSS) score and modified Rankin scale, and various parameters of oxidative stress (Table 4). The analysis was conducted separately for patients with coronary artery disease (CAD-AIS group) and those without it (AIS group).

In AIS patients with CAD (CAD-AIS group), positive correlations were observed between stroke severity measures (NIHSS and mRS) and atherogenic lipoprotein subfractions (Table 4). Specifically, NIHSS scores showed a borderline significant positive correlation with IDLC, while mRS scores were positively correlated with atherogenic subfractions of the lipoprotein spectrum, such as LDL2, LDL3–7, and IDLC.

In AIS patients, our results revealed significant negative correlations between the activities of antioxidant enzymes (catalase and GPx) and NIHSS and mRS scores. Surprisingly, total cholesterol also showed a significant negative correlation with mRS.

## 4. Discussion

The findings from our study underscore the potential role of oxidative stress and disturbed lipid metabolism in acute ischemic stroke patients (AIS) with coronary artery disease. The results of our study provide valuable insights into the effects of treatment for coronary artery disease (CAD) on various biochemical markers and their implications for cardiovascular health. The study headlines are included in Table 5.

### 4.1. Homocysteine and Triacylglycerols

One of the primary findings is that despite improvements in the lipid profile, the treatment for CAD does not seem to have a significant effect on the levels of homocysteine.

Homocysteine has long been recognized as an important risk factor for cardiovascular diseases. Elevated homocysteine levels are associated with an increased risk of atherosclerosis and coronary heart disease [23,24]. However, the relationship between homocysteine and AIS in CAD patients is complex and influenced by various factors, including genetic predisposition and impaired folate metabolism [25,26]. Elevated homocysteine levels found in CAD-AIS patients can promote inflammation and endothelial dysfunction, leading to a higher risk of thrombosis [27].

In contrast to homocysteine levels, this study also highlights the persistence of significantly higher triacylglycerol levels in AIS patients without CAD compared to CAD-AIS patients. This finding suggests a potential link between higher TAG levels and disturbances in lipid metabolism. Disturbances in lipid metabolism have long been associated with the development of atherosclerosis and cardiovascular diseases [28]. Elevated TAG levels might contribute to the accumulation of atherogenic lipoproteins, promoting inflammation and plaque formation within the arteries [29]. Clinical trials have established that elevated TAG levels can serve as a marker for residual cardiovascular risk in patients undergoing intensive lipid-lowering therapy targeted at reducing low-density lipoprotein cholesterol (LDL-C) [30]. This highlights the significance of TAG levels beyond traditional lipid measurements and emphasizes their role in risk assessment. This study’s findings align with this understanding, as AIS patients with higher TAG levels may be at an increased risk of cardiovascular events, warranting closer monitoring and potentially more aggressive management.

Our results emphasize the importance of serum homocysteine and TAG levels in assessing cardiovascular risk and treatment efficacy in patients with acute ischemic stroke and coronary artery disease. Elevated TAG levels in the AIS group may reflect disturbances in lipid metabolism and contribute to residual cardiovascular risk, while higher homocysteine levels in the CAD-AIS group could underscore the limitations of current coronary artery disease treatments. The implications of these findings might extend to potential modifications in therapeutic approaches, risk assessment strategies, and the need for continued research into understanding the intricate interplay between lipid metabolism, homocysteine levels, and cardiovascular health.

### 4.2. Age and Gender of Patients

The results of our study illuminate intriguing patterns within the context of acute ischemic stroke (AIS) and its correlation with coronary artery disease (CAD). Notably, patients in the AIS group with coexisting CAD were found to be significantly older (*p* < 0.001) in comparison to those without CAD when experiencing the AIS event. Furthermore, a compelling association emerges from our findings, revealing that a significantly higher proportion of individuals in the CAD-AIS group had previously suffered from another episode of AIS, as opposed to participants in the AIS group without CAD. This observation aligns with the logical expectation that advanced age is often accompanied by a greater susceptibility to stroke incidents.

One plausible explanation for the observed phenomenon is the regular medical supervision and enhanced care that CAD patients receive. Patients with CAD often have ongoing medical follow-up, which includes regular monitoring of their cardiovascular health and adherence to prescribed medications. This comprehensive care approach may result in better exercise and medication adherence [31]. It is necessary to admit that the management of various “traditional” and “non-traditional” vascular risk factors, including lifestyle and psychosocial stress, may contribute to the prevention of CAD [32,33]. An extensive assessment of vascular risk factors was not performed in the current study. It limits the interpretation of our results and should be considered in future prospective studies.

These outcomes underscore the intricate relationship between age, CAD, and the occurrence of both initial and recurrent AIS events. The findings prompt us to consider age as a significant factor influencing the propensity for stroke and highlight the importance of understanding these dynamics for more effective management and prevention strategies.

The gender distribution within the study groups adds another layer of complexity to the findings. In the AIS group without CAD, the substantial imbalance in the ratio of women to men (W:M ≅ 1:3) highlights potential gender-based disparities in AIS occurrence. This ratio becomes more balanced in the CAD-AIS group, where the number of women surpasses that of men (W:M ≅ 1.5:1). This shift could indicate that the presence of CAD might modify the gender-related risk factor dynamics in the context of AIS.

The age-related differences in the onset of AIS between genders in the AIS group without CAD deserve careful consideration. The observed trend of men experiencing AIS at a younger age than women is consistent with prior research findings. The mention of estrogens as potential contributors to this phenomenon echoes the well-documented cardioprotective effects of estrogens in premenopausal women [34]. The decline in estrogen levels after puberty in women and the cardioprotective role of testosterone in men offer plausible explanations for the age-related differences in AIS onset. These hormonal factors might play a critical role in shaping the gender-specific vulnerability to AIS and could be pivotal in understanding the observed age-related disparities.

The gender-specific disparities in AIS onset and outcomes, influenced by hormonal factors and treatment effects, offer important avenues for future research. Understanding these complexities is essential for developing targeted interventions that consider both gender and comorbidities, ultimately improving stroke prevention and management strategies.

### 4.3. Lipoprotein Subfractions

In this study, we did not find any significant differences in lipoprotein subfractions between the patient groups, which may be attributed to the hypolipemic treatment received by patients with coronary artery disease. Despite this lack of significant difference, we observed a significant positive correlation between the levels of atherogenic lipoprotein subfractions (IDLC, LDL2, and LDL3–7) and functional outcomes as well as disability levels (measured by mRS) in stroke patients with CAD. The presence of a positive correlation in CAD-AIS patients suggests that higher levels of certain lipoprotein subfractions could be associated with worse functional outcomes and increased disability levels in these individuals. This finding implies that the lipid profile and the composition of lipoproteins may play a role in the recovery and disability outcomes in stroke patients with CAD despite the absence of statistically significant differences between the groups. Targeting and modifying these lipoprotein subfractions through personalized treatment strategies might be beneficial in improving functional outcomes and reducing disability levels in this patient population.

In contrast, we did not observe a similar impact of these subfractions on AIS patients without CAD. This suggests that in the absence of CAD, the relationship between these specific lipid subfractions and stroke severity may not be significant. However, Zhou et al. [12] found that small dense LDL3–7 subfractions could represent an independent risk factor for AIS and that their levels were strongly associated with AIS severity and poor functional outcomes. However, they did not differentiate between AIS patients with or without CAD.

### 4.4. Oxidative Stress Markers

Our results indicate some variations in oxidative stress marker levels between the two groups, CAD-AIS and AIS. However, upon statistical analysis, all of these differences were found to be not statistically significant at the conventional *p*-value of <0.05. Although the results did not reach statistical significance, it is important to interpret the findings cautiously, as some parameters (lipoperoxides, protein carbonyls, and activity of paraoxonase 1) demonstrated *p*-values that were very close to the significance level. The lack of statistically significant differences in oxidative stress markers between the two groups could be attributed to several factors. First, the relatively small sample size might have limited this study’s statistical power to detect smaller differences that could exist between the groups. Second, the time point of measurement within 24 h after the acute ischemic stroke might not fully capture the dynamic changes in oxidative stress levels that could occur during the course of stroke recovery and progression. Despite the absence of statistical significance, the observed trends in certain oxidative stress markers could indicate subtle underlying differences in oxidative stress responses between patients with and without coronary artery disease following an acute ischemic stroke.

However, it is crucial to acknowledge that oxidative stress is a multifaceted phenomenon influenced by various factors, including inflammatory processes, lifestyle choices, and dietary habits.

One notable factor contributing to oxidative stress is smoking, a well-established risk factor for cardiovascular diseases. Haj Mouhamed et al. [35] recognized the profound impact of smoking on paraoxonase 1 (PON1) activity—a polymorphic enzyme linked to oxidative stress. It is widely known that smoking adversely affects PON1 activity, potentially exacerbating oxidative stress levels. This connection emphasizes the importance of considering lifestyle choices, such as smoking cessation interventions, in the comprehensive management of patients at risk for ischemic stroke and CAD.

Conversely, exercise has been associated with higher PON1 activity [36], which may contribute to a reduction in oxidative stress levels. Incorporating regular exercise regimens into preventive strategies could be a promising avenue for managing oxidative stress in individuals vulnerable to ischemic stroke and CAD.

In discussing the interplay of dyslipidemia, oxidative stress, and clinical outcomes, it is imperative to recognize the broader context of vascular risk factors. While our study focused on specific lipid parameters and oxidative stress markers, the influence of additional factors, including lifestyle choices and psychosocial stress, should not be overlooked. A comprehensive assessment of these factors was beyond the scope of the current study.

### 4.5. Positive Correlations between mRS and Lipoprotein Subfractions LDL2, LDL 3–7, and IDLC in CAD-AIS Patients

These correlations suggest a potential role of these lipoprotein subfractions in influencing stroke severity within this specific patient subgroup. This finding is in agreement with previously published results reporting that small dense LDL-cholesterol levels were strongly associated with AIS severity and poor functional outcomes [12]. These correlations point to possible shared mechanisms between vascular events in coronary and cerebral arteries. These lipoprotein subfractions are known players in atherosclerosis development, and their connection to stroke severity highlights their role in exacerbating both CAD and stroke outcomes [37,38].

Considering potential confounders like age, gender, and medications is essential for accurate interpretation, possibly through effects on atherosclerotic plaque formation, thrombosis, or inflammation.

Clinically, these findings suggest the potential use of these lipoprotein subfractions as biomarkers to assess stroke severity in CAD-AIS patients. This could aid risk stratification and tailored treatment strategies.

### 4.6. Negative Correlations Were Found between Antioxidant Enzyme Activities and mRS in the AIS Group

The observed negative correlation in the AIS group without CAD between antioxidant enzyme activities (catalase, glutathione peroxidase) and stroke severity, as well as with functional outcome (mRS), suggests that higher levels of antioxidant defenses might be associated with reduced neurological deficits and improved recovery. It is possible that increased antioxidant enzyme activities might offer neuroprotection by mitigating oxidative stress-induced cellular damage and reducing inflammation in the ischemic brain.

### 4.7. Negative Correlation between Total Cholesterol Levels and mRS in the AIS Group

The discovery of a significant negative correlation between total cholesterol levels and the modified Rankin Scale (mRS) scores among AIS patients without CAD carries substantial implications for our understanding of the intricate relationship between cholesterol and post-stroke outcomes. This result aligns with the previously published findings, collectively highlighting a complex interplay between cholesterol levels and stroke severity [39,40]. These converging results contradict the conventional belief about the role of cholesterol as a risk factor for cardiovascular diseases and call for a reevaluation of its potential impact on post-stroke recovery. The congruence between the present study’s findings and those published previously [39,40] strengthens the credibility of the observed negative correlation.

The assertion that higher cholesterol levels might favor the development of minor strokes, as indicated by Olsen et al. [39], provides a novel perspective on cholesterol’s potential impact. The suggestion that patients with lower cholesterol levels might experience major strokes challenges our understanding of stroke etiology and warrants an exploration of the underlying mechanisms connecting cholesterol to stroke severity.

Several hypotheses could potentially explain the observed negative correlation between total cholesterol levels and mRS scores in AIS patients. One possibility is that lower cholesterol levels might lead to reduced neuroprotection, impacting the brain’s ability to recover after ischemic injury. Cholesterol is a vital component of cell membranes and plays a crucial role in maintaining membrane integrity, neuronal function, and synaptic plasticity. Low cholesterol levels might negatively affect these processes, leading to impaired neurological recovery after ischemic injury. Another hypothesis revolves around cholesterol’s role in inflammation. Cholesterol is known to interact with the immune system and modulate inflammatory responses. Lower cholesterol levels could potentially lead to dysregulated immune reactions that impact tissue repair and regeneration after stroke. Furthermore, cholesterol is a precursor for various signaling molecules, including neurosteroids, which play a role in neuroprotection and neurogenesis. Reduced cholesterol levels might limit the availability of these neuroprotective molecules, impacting the brain’s ability to recover and adapt following ischemic insult.

The finding of a significant negative correlation between total cholesterol levels and mRS scores in AIS patients opens up new avenues for research and clinical consideration. It challenges the traditional narrative of cholesterol as purely detrimental and suggests that there might be a delicate balance between cholesterol’s role in cardiovascular health and its potential benefits in neurological recovery. These findings also underscore the need for personalized medicine approaches in stroke care.

## 5. Conclusions

The presented results shed light on the interplay between various biomarkers and their implications for cardiovascular risk in AIS patients with coronary artery disease (CAD-AIS) and acute ischemic stroke patients without the disease (AIS). Our study highlights the potential importance of antioxidant enzyme activities and lipoprotein subfractions in the context of AIS in patients with CAD. Targeting atherogenic lipoprotein subfractions through lifestyle modifications and pharmacological interventions may hold promise in mitigating stroke severity and improving functional outcomes in this high-risk population. However, further research is warranted to explore whether interventions aimed at modifying lipoprotein profiles could indeed translate into improved clinical outcomes in this context.

Moreover, the discovery of a significant negative correlation between total cholesterol levels and mRS scores among acute ischemic patients presents a thought-provoking departure from conventional wisdom. This study prompts a reevaluation of cholesterol’s role in stroke recovery and highlights the complexity of its influence on neurological outcomes. As researchers delve deeper into the mechanisms at play, the potential for novel therapeutic strategies targeting cholesterol in stroke management emerges, holding promise for improving patient outcomes and quality of life.

Despite notable improvements in lipid profiles resulting from coronary artery disease (CAD) treatment, our findings suggest a lack of significant impact on homocysteine levels. This underscores the need for continued investigation into the complexities of CAD management and its broader implications for biochemical markers associated with stroke.

A pivotal discovery in our study is the potential association between higher levels of antioxidant defenses and reduced neurological deficits, hinting at improved recovery. Increased antioxidant enzyme activities may confer neuroprotection by mitigating oxidative stress-induced cellular damage and reducing inflammation in the ischemic brain. These insights propose promising avenues for further research and highlight the potential clinical implications of enhancing antioxidant defenses in stroke patients.

To validate and build upon these findings, further investigation with a larger sample size is imperative. Future studies should prioritize investigating the impact of lifestyle factors such as diet, exercise, and smoking on acute ischemic stroke and cardiovascular diseases. Understanding the complex relationships among lipid profiles, oxidative stress markers, and lifestyle choices will provide a comprehensive foundation for developing targeted interventions.

## Figures and Tables

**Table 1:** Therapy and characteristics of the lipid profile in sera of patients with coronary artery disease and without it within 24 h after AIS.

Parameter	CAD-AIS	AIS	*p*
N (count)	37	27	
Age (years)	77.8 1± 14.41	63.89 ± 13.83	<0.001
TC (mmol/L)	3.89 ± 0.93	5.00 ± 1.33	<0.001
VLDL (mmol/L)	25.91 ± 12.09	34.86 ± 16.82	0.049
LDL (mmol/L)	2.70 ± 0.85	3.63 ± 1.33	0.001
HDL (mmol/L)	1.17 ± 0.29	1.19 ± 0.36	0.762
TAG (mmol/L)	0.90 (0.7–1.37)	1.26 (0.85–2.48)	0.023
HC (mmol/L)	16.88 ± 7.72	12.97 ± 5.50	0.037
NIHSS	5 (3–10.5)	5 (3–10.5)	0.983
mRS	3 (2–5)	3 (2–4)	0.499
Previous stroke	18 (48.6%)	5 (18.5%)	0.013
Antihypertensive therapy	32 (86.5%)	15 (55.6%)	0.006
Antidiabetic therapy	10 (27.0%)	8 (29.6%)	0.819
Antithrombotic therapy	19 (51.4%)	8 (29.6%)	0.082
Anticoagulation therapy	3 (8.1%)	1 (3.7%)	0.472
Lipid-lowering therapy	18 (48.6%)	4 (14.8%)	0.005

Results are expressed as the mean ± SD or the median (25th and 75th percentiles). CAD-AIS—the group of patients with coronary artery disease within 24 h after the acute ischemic stroke; AIS—the group of patients within 24 h after the acute ischemic stroke without coronary artery disease; TC—total cholesterol; VLDL—very low-density lipoproteins; LDL—low-density lipoproteins; HDL—high-density lipoproteins; TAG—triacylglycerols; HC—homocysteine; NIHSS—National Institutes of Health Stroke Scale; mRS—modified Rankin scale.

**Table 2:** Lipoprotein subfractions in the sera of patients with coronary artery disease and without the disease within 24 h after AIS.

Parameter	CAD-AIS	AIS	*p*
large HDL (mg/dL)	17.09 ± 5.54	14.35 ± 8.52	0.209
imHDL (mg/dL)	21.55 ± 4.92	23.30 ± 5.04	0.243
small HDL (mg/dL)	4.00 (2.00–6.00)	5.00 (4.00–6.00)	0.115
LDL1 (mg/dL)	38.68 ± 11.05	43.91 ± 18.32	0.258
LDL2 (mg/dL)	18.50 (10.50–25.25)	23.00 (13.00–37.25)	0.148
LDL3–7 (mg/dL)	0.00 (0.00–2.25)	1 (0.00–6.00)	0.421
IDLA (mg/dL)	12.36 ± 4.55	14.41 ± 7.32	0.272
IDLB (mg/dL)	7.00 ± 3.24	7.09 ± 3.02	0.924
IDLC (mg/dL)	14.82 ± 4.36	17.23 ± 5.83	0.128

Results are expressed as the mean ± SD or the median (25th and 75th percentiles). CAD-AIS—the group of patients with coronary artery disease (CAD) within 24 h after the acute ischemic stroke; AIS—the group of patients within 24 h after the acute ischemic stroke without CAD; HDL—high-density lipoproteins; LDL—low-density lipoproteins; IDL—intermediate-density lipoproteins.

**Table 3:** Oxidative stress markers in plasma of patients within 24 h after an acute ischemic stroke.

Parameter	CAD-AIS	AIS	*p*
TEAC (mmol/L)	1.64 (0.85–2.35)	1.12 (0.41–1.95)	0.131
Lipoperoxides (nmol/mL)	36.41 (25.93–70.59)	68.48 (30.13–81.55)	0.060
Isoprostanes in urine (ng/mol crea)	34.56 (24.95–65.16)	63.04 (26.414–163.66)	0.160
Protein carbonyls (mol/mg)	0.28 (0.17–0.37)	0.16 (0.09–0.30)	0.072
SOD (U/mg Hb)	684.44 ± 90.31	711.21 ± 62.26	0.233
GPx (U/mg Hb)	21.07 ± 9.04	24.50 ± 9.65	0.183
Catalase (µkat/mg Hb)	4.58 ± 0.92	4.50 ± 1.30	0.647
PON1 (U/mL)	83.39 ± 28.03	101.03 ± 38.43	0.051

Data for continuous variables are expressed as the mean ± SD or the median (25th and 75th percentiles). CAD-AIS—the group of patients with coronary artery disease within 24 h after the acute ischemic stroke; AIS—the group of patients within 24 h after the acute ischemic stroke without coronary artery disease; TEAC—trolox equivalent antioxidant capacity of plasma; SOD—superoxide dismutase; GPx—glutathione peroxidase; PON1—paraoxonase 1; crea—creatinine; Hb—hemoglobin.

**Table 4:** Significant or borderline Spearman correlations in stroke patients with coronary artery disease (CAD-AIS) and without it (AIS).

CAD-AIS				AIS			
		r	*p*			r	*p*
NIHSS	IDLC	0.410	0.058	NIHSS	Catalase	−0.430	0.044
mRS	LDL2	0.464	0.030	mRS	Catalase	−0.523	0.015
	LDL3–7	0.427	0.030		GPx	−0.486	0.048
	IDLC	0.427	0.047		TC	−0.448	0.025

r—Spearman correlation coefficient; NIHSS—National Institutes of Health Stroke Scale; mRS—modified Rankin Scale; GPx—glutathione peroxidase; TC—total cholesterol; IDLC—intermediate-density lipoprotein subfraction C; LDL—low-density lipoproteins; LDL3–7—small, dense, low-density lipoprotein subfractions.

**Table 5:** Study headlines.

− Treatment for CAD improves lipid profile but not homocysteine levels
− Higher TAG levels are confirmed to be an important risk factor for cardiovascular events
− Atherogenic lipoprotein subfractions could be a potential marker of severity of cardiovascular events in patients with atherosclerosis
− The traditional narrative of high cholesterol as an ultimate risk factor for cardiovascular diseases needs to be disputed, at least with respect to neurological outcomes

## Data Availability

The data presented in this study are available on request from the corresponding author.
